# K Index is a Reliable Marker of Intrathecal Synthesis, and an Alternative to IgG Index in Multiple Sclerosis Diagnostic Work-Up

**DOI:** 10.3390/jcm8040446

**Published:** 2019-04-02

**Authors:** Ilaria Crespi, Domizia Vecchio, Roberto Serino, Elena Saliva, Eleonora Virgilio, Maria Giovanna Sulas, Giorgio Bellomo, Umberto Dianzani, Roberto Cantello, Cristoforo Comi

**Affiliations:** 1 Laboratory of Clinical Biochemistry, Department of Health Sciences, AOU Maggiore della Carità, University of Piemonte Orientale, corso Mazzini 18, 28100 Novara, Italy; ilaria.crespi@maggioreosp.novara.it (I.C.); serinor@libero.it (R.S.); ellysally.es@gmail.com (E.S.); mgiovanna.sulas@gmail.com (M.G.S.); bellomo.giorgio@gmail.com (G.B.); umberto.dianzani@med.uniupo.it (U.D.); 2 Department of Transational Medicine, Institute of Neurology, AOU Maggiore della Carità, University of Piemonte Orientale, corso Mazzini 18, 28100 Novara, Italy; domizia.vecchio@gmail.com (D.V.); virgilioeleonora88@gmail.com (E.V.); roberto.cantello@med.uniupo.it (R.C.); 3 Interdisciplinary Research Center of Autoimmune Diseases (IRCAD), Department of Health Sciences, University of Piemonte Orientale, 28100 Novara, Italy

**Keywords:** cerebrospinal fluid, diagnosis, immunoglobulins

## Abstract

The K free light chain (K) index has been suggested as a reliable marker of intrathecal synthesis, despite the 2017 McDonald criteria for multiple sclerosis (MS) suggesting to “interpret with caution positive immunoglobulin G (IgG) index when testing for oligoclonal bands (OB) is negative or not performed”. The aim of this study was to compare the performance of K and IgG indexes for MS diagnosis and OB detection in a cohort of Italian patients. We enrolled 385 patients (127 MS, 258 non-MS) who had cerebrospinal fluid (CSF) analysis, including isoelectric focusing (IEF), to detect OB in the diagnostic work-up. Albumin, IgG and free light chains were measured by nephelometry and used to calculate IgG and K indexes. Although the two markers were highly related (*r* = 0.75, *r*^2^ = 0.55, *p* < 0.0001), the K index showed greater sensitivity and negative predictive value (versus the IgG index) for OB detection (97% versus 48% and 97% versus 71%) and MS diagnosis (96% versus 50% and 98% versus 78%). These results support K index (and not IgG index) as a first-line marker for MS, followed by IEF, according to a sequential testing approach in CSF analysis.

## 1. Introduction

Multiple sclerosis (MS), a chronic inflammatory demyelinating disease of the central nervous system, is diagnosed according to McDonald criteria with the exclusion of other differentials [1]. Since the first version of the criteria, cerebrospinal fluid (CSF) analysis has been included to evaluate intrathecal antibody synthesis as a supportive finding [2]. The 2017 revision upgraded the role of CSF analysis to diagnose MS in subjects who presented a single episode, instead of a second clinical event and/or increased lesion load [3]. The gold standard for CSF analysis is isoelectric focusing (IEF) to detect CSF oligoclonal bands (OB) of immunoglobulin (Ig) G. Historically, intrathecal synthesis of IgG has been detected as an elevated immunoglobulin G (IgG) or Link index, calculated as the ratio of CSF/serum IgG to CSF/serum albumin [4]. Recently, measures of free light chains (FLC) by nephelometry have been proposed as an alternative method to IgG index and OB detection by IEF [5]. Despite the role of lambda (λ) FLC remains controversial, there is robust evidence for kappa (K) FLC [6,7]. The rationale of this biochemical approach is to detect intrathecal KFLC with high sensitivity as evidence of B-cell activity within the CSF. As for IgG, the ratio of CSF/serum KFLC may be corrected by the ratio of CSF/serum albumin to obtain the KFLC (or K) index [6]. In this context, the K index is proposed as a highly sensitive first-line test for intrathecal synthesis of Ig followed by IEF, in a sequential approach [8] similarly to thyroid hormones testing [9].

It must be underlined that the 2017 McDonald criteria includes the recommendation to “use caution with positive IgG index when testing for OB is negative or not performed” [3], and discussion of the actual role of KFLC versus OB testing has not reach a definite agreement to date [10]. However, a sequential method has been recently considered as reasonable [8,9,10,11]. The aims of this study were to confirm the role of the K index in CSF screening for MS and compare its performances to that of the IgG index and OB in a large cohort of Italian patients.

## 2. Experimental Section

A total of 385 patients was consecutively enrolled in the study and underwent lumbar puncture (LP) in their diagnostic work-up for CSF biochemistry and IEF (according to the requesting neurologist) from January 2014 to January 2018, which included those patients described by Crespi [8]. The mean age of patients was 48 years (standard deviation, SD ± 18). Diagnosis was prospectively collected by a different neurologist and compared with the initial clinical suspicion. Of the 385 patients, 127 (33%) were diagnosed with MS according to the 2017 McDonald criteria [3], 117 (30%) with other neurological inflammatory diseases (ID: inflammatory neuropathies, acute demyelinating encephalomyelitis, systemic autoimmune disorders with central nervous system involvement) and 141 (37%) with non-inflammatory neurological diseases (NID: amyotrophic lateral sclerosis, dementia, non-inflammatory neuropathies, tumors). Patients signed an informed consent form for both diagnostic and research purposes at the time of the LP. The study was approved by the ethical committee of the University Hospital of Novara (reference no. CE1804). This study did not include identifiable patient data.

The OB detection was achieved by isoelectric focusing and immunofixation (Hydragel 9 CSF Isofocusing; Sebia, Bagno a Ripoli, FI, Italy) on an agarose electrophoresis system (Sebia Hydrasys). Two independent operators, blind to the diagnosis, evaluated the results and classified the OB pattern into five different types: (1) no bands in CSF and serum; (2) oligoclonal IgG in CSF but not in serum; (3) IgG bands in both CSF and serum, with additional bands in CSF; (4) identical oligoclonal bands in CSF and serum; (5) monoclonal IgG pattern in both CSF and serum. Types 2 and 3 are associated with intrathecal immunoglobulin synthesis [12]. Serum and CSF albumin, Ig, λ and KFLC were measured by nephelometric assays using BN II System (Siemens Healthcare Diagnostics Products GmbH, Marburg, Germany) according to methods used in a previous paper [8]. We calculated IgG (or Link) and KFCL (or K) indexes as well as Reiber-modified parameters (IgG loc and K loc) according to published formulas [10,13]. 

Sensitivity (%), specificity (%), likelihood ratio for positive test, likelihood ratio for negative test and positive and negative predictive values (%) were measured using the Bayesian calculator developed by SIPMEL (Società Italiana di Patologia Clinica e Medicina di Laboratorio) [14].

Statistical analyses were performed using VassarStats (website for statistical computation) [15]. The t test for two independent samples was used for group comparison with equal and/or unequal sample variances. The difference between three or more samples was calculated using one-way ANOVA. Qualitative variables, e.g., the frequency of KFLC intrathecal synthesis among the disease groups, were compared with χ^2^ test. Spearman’s coefficient was used for correlation analysis. Receiver Operating Characteristic curve analysis was performed using XLSTAT statistical package (Addinsoft Inc., Long Island City, NY, USA).

## 3. Results

### 3.1. Markers of Intrathecal Synthesis in MS

**Figure 1:**
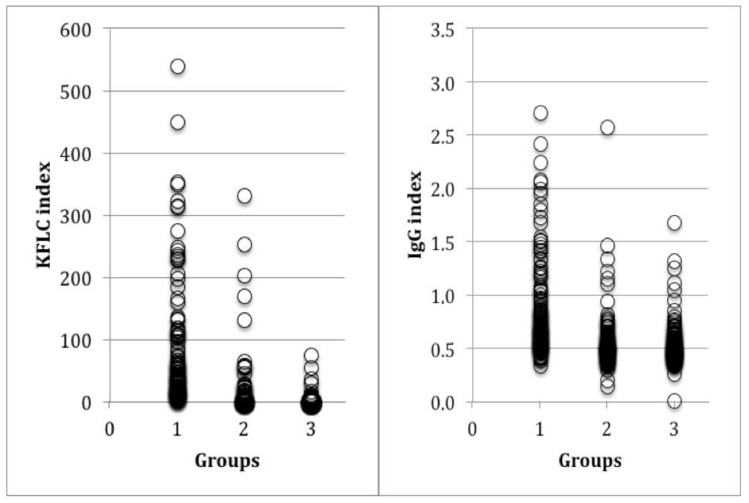
Kappa (K) (**left**) and immunoglobulin G (IgG) (**right**) indexes in patients with multiple sclerosis (MS) in group 1, inflammatory diseases (ID) in group 2 and non-inflammatory diseases (NID) in group 3.

The 127 MS patients had a median K index of 72.9 ± a SD of 87.9 that was significantly higher than that displayed by the other 258 patients (12.7 ± 48.9; *p* < 0.0001), including both ID (23.5 ± 70.7; *p* < 0.0001) and NID (3.8 ± 8.4; *p* < 0.0001), as shown in Figure 1 and Table 1. The K index was >5 in 96.1% MS, 19.4% non-MS, 33.3% ID, and 7.8% NID patients. 

The IgG index values were higher in MS (0.86 ± 0.5) than in the other patients (0.50 ± 0.23; *p* < 0.0001). The IgG index was >0.7 in 59.1% MS, 15.1% non-MS, 17.9% ID, and 12.8% NID patients. 

OB types 2 and 3 were detected in 123 (96.8%) MS patients and 44 (17%) patients with other neurological disorders (35 ID and nine NID patients).

Analysis of MS, ID and NID patients showed that the K index was different in the three groups (ANOVA *p* < 0.001) whereas the IgG index was not able to differentiate ID from NID (the Tukey test was not significant). In addition, a correlation was confirmed between IgG and K indexes in all patients (*n* = 385: *r* = 0.75, *r*^2^ = 0.55, *p* < 0.0001) and in each group (Supplementary material Table S1). 

### 3.2. Comparisons and Diagnostic Performances of K and IgG Indexes

**Figure 2:**
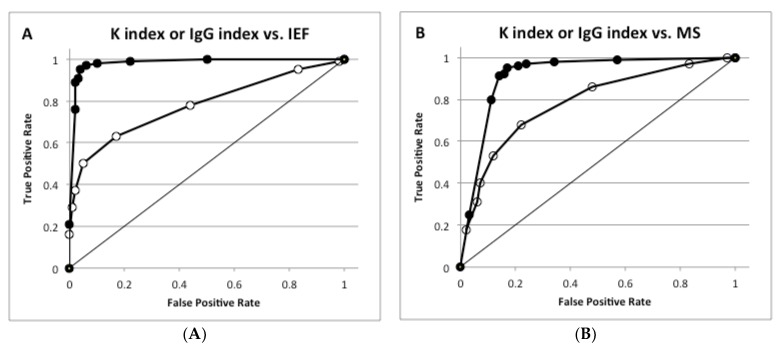
Receiver operating characteristic (ROC) analysis for K and IgG indexes in predicting OB detection and diagnosing multiple sclerosis. (**A**) K index or IgG index vs. isoelectric focusing (IEF); (**B**) K index or IgG index vs. MS.

To compare the ability of the K and IgG indexes to predict intrathecal synthesis, their correlation with OB and MS diagnosis was analyzed by receiver operating characteristic (ROC) analysis. The K index showed a better area under curve (AUC) (0.981) than the IgG index (0.778) to predict OB (Figure 2A) and to diagnose MS (AUC of the K index 0.949 vs. AUC of the IgG index 0.789, Figure 2B).

In panel A, ROC analysis was performed for OB (types 2 and 3 by IEF) versus the absence of OB (types 1, 4 and 5); AUC was 0.778 for IgG (open symbols) and 0.981 for K index (closed symbols). In panel B, ROC analysis was performed for MS patients versus non-MS patients; AUC was 0.789 for IgG (open symbols) and 0.949 for K index (closed symbols).

The diagnostic parameters of both indexes in predicting OB and diagnosing MS were then considered. In detecting OB, the K index (cut off value 5) showed a significantly better sensitivity, likelihood ratio for a positive test, positive and negative predictive values and efficacy compared to the IgG index (cut off value 0.7) [4]. By contrast, the specificity was similar for both indexes. In diagnosing MS, the K index exhibited higher sensitivity, negative predictive value and efficiency than the IgG index (Table 2 and Table 3).

### 3.3. Confounding Effects of Blood–Brain Barrier Permeability

The calculation of IgG and K indexes was based on an equivalent linear relationship between albumin and IgG or KFLC, irrespective of the size differences of these molecules: albumin was 69 KDa, IgG was 155 Kda, and KFLC was 22 KDa. For this reason, IgG loc and K loc were also calculated using the Reiber formula that employs a hyperbolic relationship to correct for the size and repeated analysis. The new parameters did not exhibit a better ROC curve for OB (IgG loc vs. IgG index AUC: 0.789 vs. 0.778; K loc vs. K index AUC: 0.978 vs. 0.981) or MS diagnosis (IgG loc vs. IgG index AUC: 0.801 vs. 0.789; K loc vs. K index AUC: 0.921 vs. 0.949). No significant differences were observed according to the other performance parameters (Table 2 and Table 3; supplementary material Figures S1 and S2).

## 4. Discussion

This study extends our previous analysis [8] on a larger cohort of patients and compares the diagnostic performances of K and IgG indexes in detecting intrathecal synthesis and diagnosing MS. 

It is widely accepted that the intrathecal synthesis of IgG is mirrored by the detection of OB bands in CSF (exclusively in CSF than in serum), as analyzed by IEF followed by immunoblotting [16]. These qualitative findings are generally associated with a positive IgG index (above 0.7) and a positive K index (above 5 in ourlaboratory setting) [8]. Since IEF for OB detection is the gold standard approach in MS diagnosis, this study compared the ability of IgG and K indexes to detect OB and diagnose MS.

Despite the two markers correlating in both MS and non-MS patients, they showed different diagnostic performances according to ROC analyses. In fact, the K index displayed better AUC values, not only in predicting OB but also in diagnosing MS. Consequently, the performances of a sequential test to detect intrathecal synthesis in MS was evaluated using a quantitative marker, followed by IEF when elevated. The K index showed higher sensitivities in predicting OB and diagnosing MS. The K index identified 96.1% of the MS patients whereas the IgG index only identified 59.1%. Specificity was comparable for the two indexes. It was concluded that the K index was more efficient than the IgG (Link) index as a quantitative test for intrathecal synthesis. 

Secondly, the ability of the IgG and K indexes to discriminate ID and NID patients were compared. The IgG index was not able to differentiate the two conditions, whereas the K index was elevated in 33.3% ID and 7.8% NID patients. Moreover, OB were detectable in 29.9% of ID and 6.4% of NID patients; in these groups, cases with types 2 or 3 IEF also had an elevated KFLC index (data not shown). Thus, it can be concluded that the K index may help to differentiate ID from NID patients better than the IgG index. 

Finally, this study was unable to demonstrate that the correction of the IgG or K indexes according to Reiber’s formula may ameliorate the diagnostic power, which supports the value of the method used in CSF analysis.

## 5. Conclusions

These results confirmed our previous proposal to use the K index as a highly sensitive and easy to detect first-line marker in CSF analysis for intrathecal synthesis. If the K index is elevated, this first analysis should be followed by IEF. By contrast, the IgG index should not be chosen for this role since it showed lower diagnostic performances. This sequential testing may be an optimal procedure for diagnosing MS with accurate performance and low cost. Of note, this sequential testing is being used in our clinical practice according to recent evidence [17,18,19] 

## Figures and Tables

**Table 1:** Prevalence of elevated K and IgG indexes and oligoclonal bands (OB) types 2 and 3 among patients with multiple sclerosis (MS), inflammatory diseases (ID) and non-inflammatory diseases (NID).

Parameters	MS (*n*: 127)	Non-MS (*n*: 258)	ID (*n*: 117)	NID (*n*: 141)
IgG index > 0.7 (*n*, %)	75/127 (59.1%)	39/258 (15.1%)	21/117 (17.9%)	18/141 (12.8%)
K index > 5 (*n*, %)	122/127 (96.1%)	50/258 (19.4%)	39/117 (33.3%)	11/141 (7.8%)
OB (*n*, %)	123/127 (96.8%)	44/258 (17%)	35/117 (29.9%)	9/141 (6.4%)

**MS:** multiple sclerosis; ID: inflammatory diseases; NID: non-inflammatory diseases, OB: oligoclonal bands; *n*: number.

**Table 2:** Diagnostic performances of IgG index, IgG loc, K index and K loc for OB detection by IEF.

	OB Detection			
	IgG Index	IgG loc	K Index	K loc
Sensitivity (%)	48.0 (41.2–54.9)	47.6 (40.6–54.7)	96.5 (93.0–98.3)	95.8 (91.8–97.8)
Specificity (%)	93.8 (90.3–96.1)	95.5 (92.0–97.4)	89.8 (85.7–92.9)	93.3 (89.4–95.8)
Likelihood ratio for a positive test	7.8 (4.8–12.6)	10.5 (5.8–19.0)	9.48 (6.7–13.5)	14.2 (8.8–22.9)
Likelihood ratio for a negative test	0.6 (0.5–0.6)	0.6 (0.5–0.6)	0.04 (0.02–0.08)	0.04 (0.02–0.09)
Positive predictive value (%)	85.1 (77.4–90.5)	89.1 (81.5–93.8)	87.4 (82.5–91.2)	91.9 (87.2–94.9)
Negative predictive value (%)	71.1 (66.2–75.5)	70.0 (64.8–74.7)	97.2 (94.4–98.7)	96.5 (93.3–98.2)
Efficiency (%)	74.4 (70.3–78.1)	74.5 (70.2–78.4)	92.7 (89.9–94.7)	94.4 (91.8–96.2)
Pre-test probability (prevalence) (%)	42.3 (37.9–46.8)	43.8 (39.2–48.6)	42.3 (37.9–48.8)	44.3 (39.6–49.0)
Pre-test odds	0.7 (0.6–0.9)	0.8 (0.7–0.9)	0.7 (0.6–0.9)	0.8 (0.7–0.96)
Post-test odds	5.7 (2.9–11.1)	8.2 (3.7–17.9)	6.9 (4.1–11.9)	11.3 (5.8–22.0)
Post-test probability (%)	85.1 (74.6–91.7)	89.1 (78.8–94.7)	87.4 (80.3–92.2)	91.9 (85.3–95.7)
Number needed to diagnose (NND)	2.4 (3.2–2.0)	2.3 (3.2–1.9)	1.2 (1.3–1.1)	1.1 (1.2–1.1)

The analysis was performed for OB (types 2 and 3 by IEF) versus absence of OB (types 1, 4 and 5 by IEF); data are reported with their 95% confidence intervals.

**Table 3:** Diagnostic performances of IgG index, IgG loc, K index and K loc for MS diagnosis.

	MS Diagnosis			
	IgG Index	IgG loc	K Index	KFLC loc
Sensitivity (%)	49.6 (42.5–56.7)	51.3 (42.4–60.1)	96.1 (90.8–98.5)	95.8 (90.5–98.2)
Specificity (%)	88.5 (84.2–92.7)	90.7 (86.6–93.7)	77.9 (72.0–83.7)	80.7 (75.5–85.0)
Likelihood ratio for a positive test	4.3 (3.1–7.0)	5.5 (3.6–8.4)	4.4 (3.1–5.9)	5.0 (3.9–6.4)
Likelihood ratio for a negative test	0.6 (0.5–0.7)	0.5 (0.4–0.7)	0.05 (0.02–0.11)	0.05 (0.02–0.12)
Positive predictive value (%)	67.7 (58.2–76.9)	71.8 (61.4–80.2)	68.2 (60.9–75.4)	69.5 (62.1–76.0)
Negative predictive value (%)	78.4 (73.3–82.9)	80.2 (75.3–84.4)	97.6 (94.5–98.8)	97.6 (94.6–98.9)
Efficiency (%)	75.8 (70.1–80.7)	78.3 (73.9–82.2)	83.9 (80.7–87.5)	85.4 (81.5–88.6)
Pre-test probability (prevalence) (%)	32.6 (28.0–37.2)	31.5 (27.0–36.3)	33.0 (28.5–38.3)	31.5 (27.0–36.3)
Pre-test odds	0.5 (0.4–0.6)	0.5 (0.4–0.6)	0.5 (0.4–0.6)	0.5 (0.4–0.6)
Post-test odds	2.1 (1.2–3.4)	2.5 (1.4–4.8)	2.1 (1.5–3.7)	2.1 (1.5–3.6)
Post-test probability (%)	67.7 (57.9–76.8)	71.8 (57.4–82.8)	68.2 (58.0–67.3)	69.5 (58.8–78.5)
Number needed to diagnose (NND)	2.6 (3.8–2.1)	2.4 (3.5–1.9)	1.5 (1.5–1.2)	1.5 (1.5–1.2)

The analysis was performed for MS patients versus non-MS patients; data are reported with their 95% confidence intervals.

## Supplementary Materials

The following are available online at https://www.mdpi.com/2077-0383/8/4/446/s1, Figure S1: KFLC loc and IgG loc in MS (group 1), ID (group 2) and NID (group 3) patients; Figure S2: Correlations between KFLC index, IgG index, KFLC loc and IgG loc in patients with multiple sclerosis; Table S1: Correlations between IgG index and KFLC index among patients with multiple sclerosis (MS), inflammatory diseases (ID) and non-inflammatory diseases (NID).

## References

[1] Polman C.H., Reingold S.C., Banwell B. (2011). Diagnostic criteria for multiple sclerosis: 2010 revisions to the McDonald Criteria. Ann. Neurol..

[2] Poser C.M., Paty D.W., Scheinberg L. (1983). New diagnostic criteria for multiple sclerosis: Guidelines for research protocols. Ann. Neurol..

[3] Thompson A., Banwell B., Barkoff F. (2018). Diagnosis of multiple sclerosis: 2017 revisions of the McDonald Criteria. Lancet Neurol..

[4] Link H. (1976). The value of cerebrospinal fluid immunoglobulin analysis in clinical neurology. Riv. Patol. Nerv. Ment..

[5] Bayart J.L., Muls N., van Pesch V. (2018). Free kappa light chains in neuroinflammatory disorders: Complement rather than substitute?. Acta Neurol. Scand..

[6] Ramsden D.B. (2017). Multiple sclerosis: Assay of free immunoglobulin light chains. Ann. Clin. Biochem..

[7] Zeman D., Kušnierová P., Švagera Z., Všianský F., Byrtusová M., Hradílek P., Kurková B., Zapletalová O., Bartoš V. (2016). Assessment of intrathecal free light chain synthesis: Comparison of different quantitative methods with the detection of oligoclonal free light chains by isoelectric focusing and affinity-mediated immunoblotting. PLoS ONE.

[8] Crespi I., Sulas M.G., Mora R., Naldi P., Vecchio D., Comi C., Cantello R., Bellomo G. (2017). Combined use of kappa free light chain index and isoelectrofocusing of cerebro-spinal fluid in diagnosing multiple sclerosis: Performances and costs. Clin. Lab..

[9] Caldarelli G., Troiano G., Rosadini D., Nante N. (2017). Adoption of TSH Reflex algorithm in an Italian clinical laboratory. Ann. Igy..

[10] Gurtner K.M., Shosha E., Bryant S.C., Andreguetto D., Murray D.L., Pittock S.J., Willrich M.A.V. (2018). CSF free light chain identification of demyelinating disease: Comparison with oligoclonal bands and other CSF indexes. Clin. Chem. Lab. Med..

[11] Schwenkenbecher P., Konen F.F., Wurster U., Jendretzky K.F., Gingele S., Sühs K.W., Pul R., Witte T., Stangel M., Skripuletz T. (2018). The persisting significance of oligoclonal bands in the dawning era of kappa free light chains for the diagnosis of multiple sclerosis. Int. J. Mol. Sci..

[12] Deisenhammer F., Bartos A., Egg R., Gilhus N.E., Giovannoni G., Rauer S., Sellebjerg F. (2006). Guidelines on routine cerebrospinal fluid analysis. Report from an EFNS task force. Eur. J. Neurol..

[13] Presslauer S., Milosavljevic D., Huebl W., Aboulenein-Djamshidian F., Krugluger W., Deisenhammer F., Senel M., Tumani H., Hegen H. (2016). Validation of kappa free light chains as a diagnostic biomarker in multiple sclerosis and clinically isolated syndrome. A multicenter study. Mult. Scler..

[14] https://www.sipmel.it/it/risorse/strumenti.

[15] http://vassarstats.net/.

[16] Gastaldi M., Zardini E., Leante R., Ruggieri M., Costa G., Cocco E., De Luca G., Cataldo I., Biagioli T., Ballerini C. (2017). Cerebrospinal fluid analysis and the determination of oligoclonal bands. Neurol. Sci..

[17] Emersic A., Anadolli V., Krsnik M., Rot U. (2019). Intrathecal immunoglocbulin synthesis: The potential value of an adjunct test. Clin. Chim. Acta.

[18] Vecchio D., Crespi I., Virgilio E., Naldi P., Campisi M.P., Serino R., Dianzani U., Bellomo G., Cantello R., Comi C. (2019). Kappa free light chains could predict early disease course in multiple sclerosis. Mult. Scler. Relat. Disord..

[19] Solaro C., Ponzio M., Moran E., Tanganelli P., Pizio R., Ribizzi G., Venturi S., Mancardi G.L., Battaglia M.A. (2015). The changing face of multiple sclerosis: Prevalence and incidence in an aging population. Mult. Scler..

