# Supplementary material for: K Index is a Reliable Marker of Intrathecal Synthesis, and an Alternative to IgG Index in Multiple Sclerosis Diagnostic Work-Up
Source: J Clin Med. 2019 Apr 2;8(4):446. doi: 10.3390/jcm8040446 (PMC6518364; doi:10.3390/jcm8040446)

Methods

Intrathecally synthesized KFLC was also calculated using a non-linear function by relating each $KFLC_{ratio}$ (CSF kFLC/serum kFLC) to its corresponding QAlb-dependent upper normal limit ($KFLC_{lim}$). $KFLC_{lim}$ has been determined in a large cohort of patients in a previous study (Presslauer), specified by the following formula: $kFLC_{lim} = 0.9358 \times QAlb^{0.6687}$.
The change of KFLC concentration in CSF was calculated as the difference between $KFLC_{ratio}$ and $kFLC_{lim}$, and finally corrected for the absolute KFLC serum concentration: $KFLC_{loc} = (KFLC_{ratio} - KFLC_{lim}) \times KFLC_{serum}$.

The same approach was employed to calculate identical parameters for intrathecally synthetized IgG (IgG_{loc}).

Table S1: Correlations between IgG index and KFLC index among patients with multiple sclerosis (MS), inflammatory diseases (ID) and non-inflammatory diseases (NID).

**Parameters**	***n***	**r**	**r²**	***p***
**All**	385	0.7469	0.5579	<0.0001
**MS**	127	0.7780	0.6053	<0.0001
**ID**	117	0.6265	0.3921	<0.0001
**NID**	141	0.6789	0.4609	<0.0001

Figure S1: KFLC loc and IgG loc in MS (group 1), ID (group 2) and NID (group 3) patients.

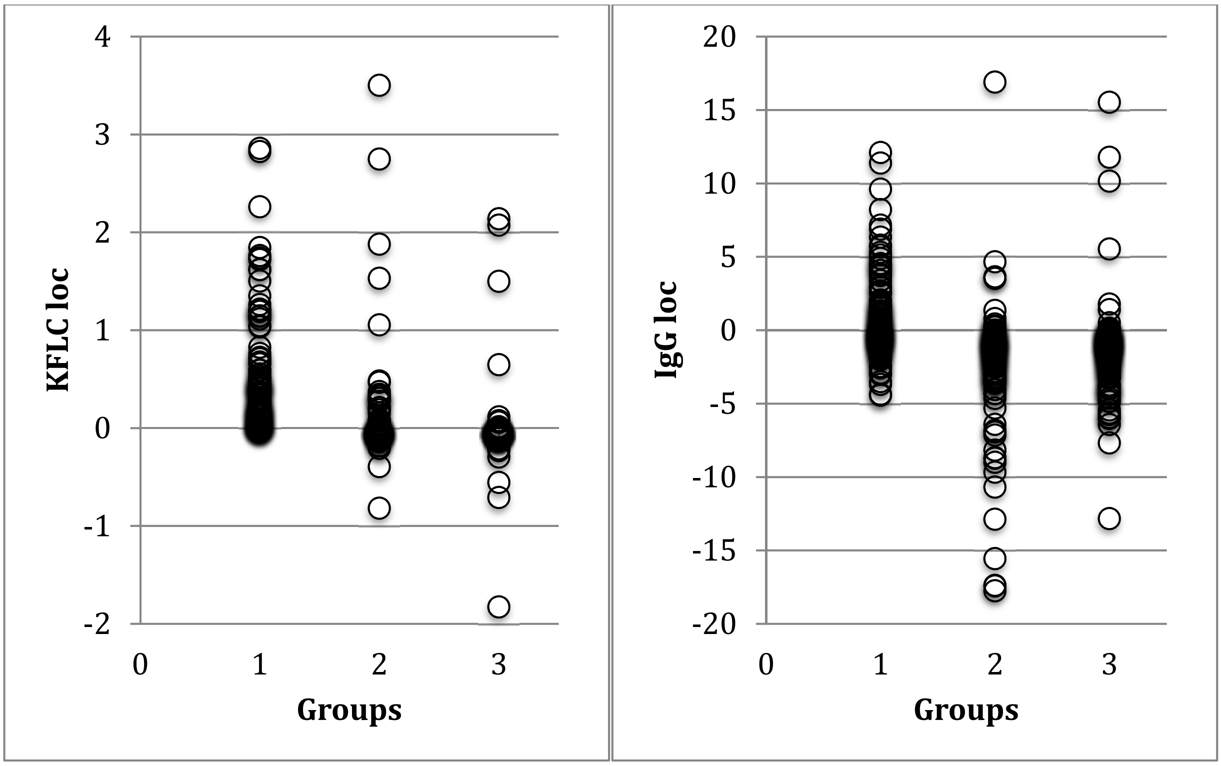

Figure S2: Correlations between KFLC index, IgG index, KFLC loc and IgG loc in patients with multiple sclerosis.

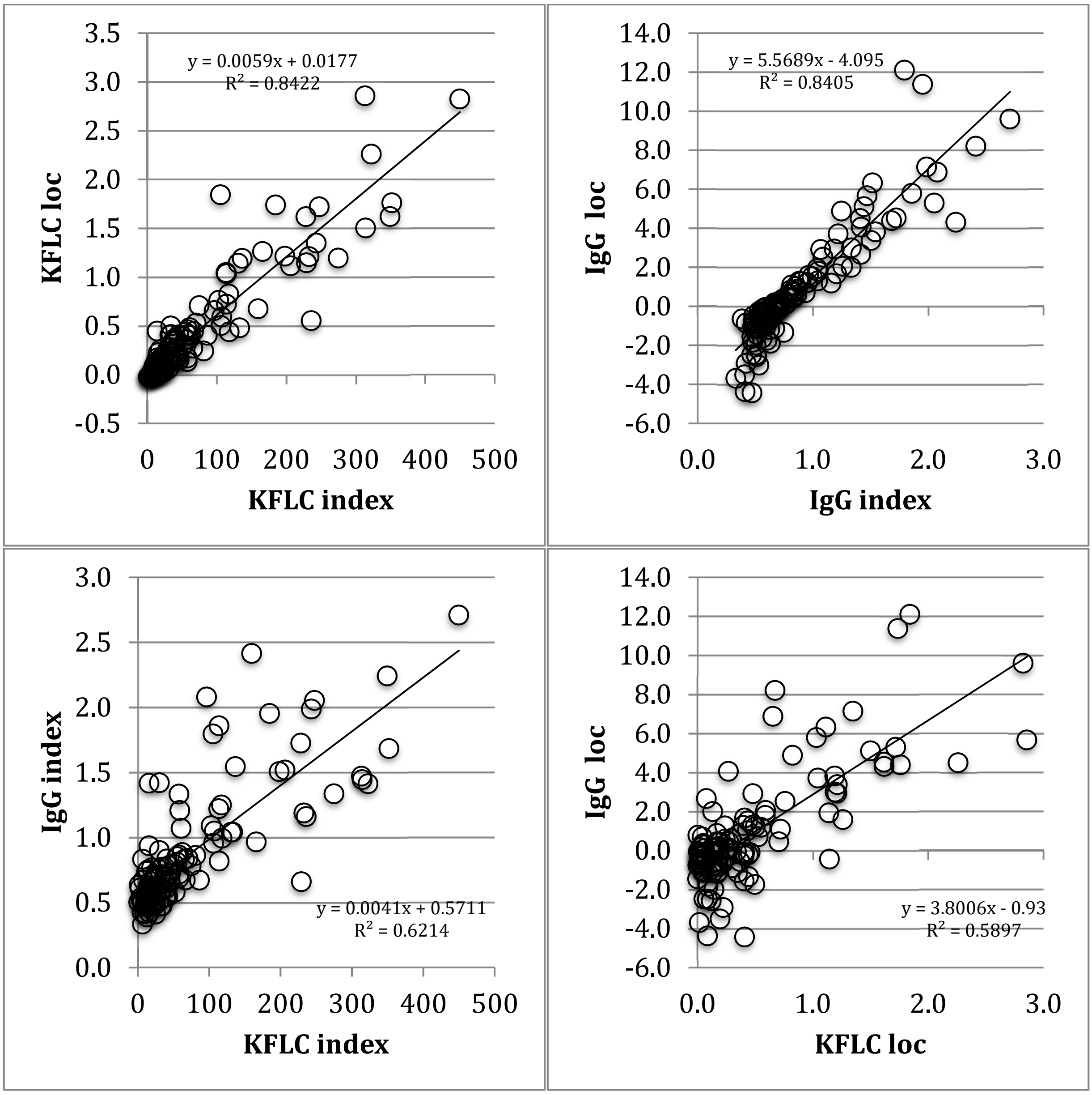

Supplement: Supplementary file 1 [file jcm-08-00446-s001.pdf]
